# Green Tea Catechin, EGCG, Suppresses PCB 102-Induced Proliferation in Estrogen-Sensitive Breast Cancer Cells

**DOI:** 10.1155/2015/163591

**Published:** 2015-12-13

**Authors:** Kimberly Mantzke Baker, Angela C. Bauer

**Affiliations:** ^1^Department of Biology, University of Indianapolis, Lilly Science Hall 30, 1400 East Hanna Avenue, Indianapolis, IN 46227, USA; ^2^Department of Biology, High Point University, Congdon 217, 833 Montlieu Avenue, High Point, NC 27268, USA

## Abstract

The persistence of polychlorinated biphenyls (PCBs) in the environment is of considerable concern since they accumulate in human breast tissue and may stimulate the growth of estrogen-sensitive tumors. Studies have shown that EGCG from green tea can modify estrogenic activity and thus may act as a cancer chemopreventive agent. In the present study, we evaluated the individual and combined effects of PCB 102 and EGCG on cell proliferation using an estrogen-sensitive breast cancer cell line MCF-7/BOS. PCB 102 (1–10 *μ*M) increased cell proliferation in a dose-dependent manner. Furthermore, the proliferative effects of PCB 102 were mediated by ER*α* and could be abrogated by the selective ER*α* antagonist MPP. EGCG (10–50 *μ*M) caused a dose-dependent inhibition of PCB 102-induced cell proliferation, with nearly complete inhibition at 25 *μ*M EGCG. The antiproliferative action of EGCG was mediated by ER*β* and could be blocked by the ER*β*-specific inhibitor PHTPP. In conclusion, EGCG suppressed the proliferation-stimulating activity of the environmental estrogen PCB 102 which may be helpful in the chemoprevention of breast cancer.

## 1. Introduction

Endocrine disrupting chemicals (EDCs) are environmental contaminants that have the ability to interfere with hormone signaling in the body. EDCs can disrupt endocrine function in a variety of ways: through enhancement or inhibition of hormone synthesis [1, 2]; through activation or antagonism of hormone receptors [3, 4]; and/or through blockade of hormone intracellular signaling [5]. A variety of substances—both natural and man-made—have been identified as EDCs, including (but not limited to) pesticides [1, 6], plasticizers (such as bisphenol A [5, 7]), pharmaceuticals [8], sunscreens [9], and triclosan (found in antibacterial soaps [10, 11]). Exposure to some EDCs is associated with negative health effects in both humans and wildlife. These negative health effects include infertility [12], intersexed conditions [12, 13], increased risk of endometriosis [14], and increased risk of certain cancers (especially hormone-sensitive cancers, like breast and prostate cancers [12]).

Xenoestrogens are a specific group of EDCs that disrupt hormone signaling by mimicking the actions of estrogen within the body. Several of the polychlorinated biphenyls (PCBs)—persistent organic pollutants found in soil, air, water, and food—are categorized as xenoestrogens, given the fact that they exert estrogenic effects through direct binding of estrogen receptors, particularly ER*α* [15–17]. A variety of human tissues—blood, adipose tissue, and milk—exhibit significant accumulation of PCBs, due to the low degradation rate and fat solubility of these estrogenic pollutants [18, 19]. The PCB concentrations detected in these tissues—in particular, in breast fat and milk—fall within the range of those found in laboratory studies to exert physiologic effects via estrogen receptors [19, 20]. As a result, many scientists have postulated a potential role for PCBs in the increased incidence of estrogen-sensitive cancers, including breast cancer. Indeed, some studies suggest that levels of specific PCBs present in the breast fat of women are positively correlated with the incidence of malignant tumors [21, 22], and certain PCBs have been shown to enhance the proliferation of estrogen-sensitive breast cancer cells* in vitro* [3, 16, 17, 20].

Recent studies indicate that certain polyphenolic compounds found in foods (green tea, red wine, chocolate, and fruits) can also act like xenoestrogens and exert biologic effects through the activation of estrogen receptors [23, 24]. However, unlike the proliferative effects of certain PCBs exerted via estrogen receptors within cancer cells, these polyphenolic compounds exert chemopreventive actions via estrogen receptors in cancer cells. Epigallocatechin gallate (EGCG), the major catechin found in green tea, is one of these chemopreventive compounds* in vitro*. EGCG has been shown to bind to both ER*α* and ER*β* [23] and to inhibit proliferation of the estrogen-sensitive MCF-7 breast cancer cell line [24]. In addition to antiproliferative effects exerted via estrogen receptors, EGCG also exerts ER-independent actions that result in inhibition of aryl hydrocarbon- (AhR-) regulated genes and induction of apoptosis [25–27].

Several epidemiologic and experimental studies have demonstrated a positive correlation between the consumption of estrogenic polyphenolic compounds and cancer prevention [28, 29]. Furthermore, some experiments have demonstrated that the proliferative effects of environmental EDCs on cancer cells can be partially or fully inhibited by cotreatment with polyphenolic compounds [30]. Such findings suggest that the detrimental health effects of EDCs, like the PCBs, could potentially be counteracted by a diet that is rich in polyphenolic, chemopreventive compounds like EGCG (found in green tea). In light of this possibility, the present study was conducted in order to determine whether EGCG can inhibit the proliferative effects of an estrogenic PCB (specifically PCB 102) on the proliferation of the estrogen-sensitive breast cancer cell line, MCF-7/BOS.

## 2. Materials and Methods

### 2.1. Chemicals and Reagents

Epigallocatechin-3-gallate (EGCG), 17*β*-estradiol (E2), and dimethylsulfoxide (DMSO) were purchased from Sigma (St. Louis, MO). 2,2′,4,5,6′-Pentachlorobiphenyl (PCB 102) was obtained from AccuStandard (New Haven, CT). The selective ER*α* antagonist 1,3-bis(4-hydroxyphenyl)-4-methyl-5-[4-(2-piperidinylethoxy)phenol]-1H-pyrazole dihydrochloride (MPP dihydrochloride) and the selective ER*β* antagonist 4-[2-phenyl-5,7-bis(trifluoromethyl)pyrazolo[1,5-a]pyrimidin-3-yl]phenol (PHTPP) were purchased from R&D Systems, Inc. (Minneapolis, MN).

### 2.2. Cell Culture

MCF-7/BOS human breast cancer cells were kindly provided by Dr. Ana Soto (Tufts University, Boston, MA). The cells were grown in Dulbecco's modified Eagle's medium (DMEM) (Hyclone, Logan, UT) supplemented with 5% fetal bovine serum (FBS) (Mediatech Inc., Manassas, VA), 100 U/mL penicillin, 100 *μ*g/mL streptomycin, and 0.25 *μ*g/mL amphotericin B (Hyclone, Logan, UT). Cells were cultured as monolayers and maintained at 37°C in a 5% CO_2_ humidified environment.

### 2.3. Cell Proliferation Assay

MCF-7/BOS cells were seeded in 12-well plates (Corning Inc., Corning, NY) at 8 × 10^4^ cells per well for 24 hours to allow the cells to attach. The culture medium was then changed to phenol red-free DMEM F12 supplemented with 5% Charcoal/Dextran treated FBS (CD-FBS) (Hyclone, Logan, UT) containing the test compounds PCB 102 (1–10 *μ*M), EGCG (10–50 *μ*M), 1 nM E2, 1 *μ*M MPP, and 1 *μ*M PHTPP dissolved in DMSO (final concentration 0.1–0.2%). In the assays with PCB 102 ± MPP, PHTPP, or EGCG the cells were cotreated for 72 hours and then harvested by trypsinization. For the EGCG ± ER antagonist assays, cells were pretreated with 1 *μ*M MPP or PHTPP for 24 hours and then treated with EGCG for an additional 72 hours before harvesting. Cell proliferation was assessed by counting with a hemocytometer and results were expressed as a percentage of the control cells (media with DMSO) on a plate-by-plate basis. In selective experiments, the trypan blue (Hyclone, Logan, UT) dye exclusion assay was used to assess cell viability.

### 2.4. Statistical Analysis

SAS (v. 9) for Windows was used for statistical analysis. Data were expressed as the mean ± standard error (SE) of 3 independent experiments performed in duplicate. Statistical differences among the groups were analyzed using one-way analysis of variance (ANOVA) followed by Dunnett's or Tukey's multiple comparison test where appropriate. A statistically significant difference was set at *P* < 0.05.

## 3. Results

### 3.1. Effect of PCB 102 on MCF-7/BOS Breast Cancer Cell Proliferation

**Figure 1:**
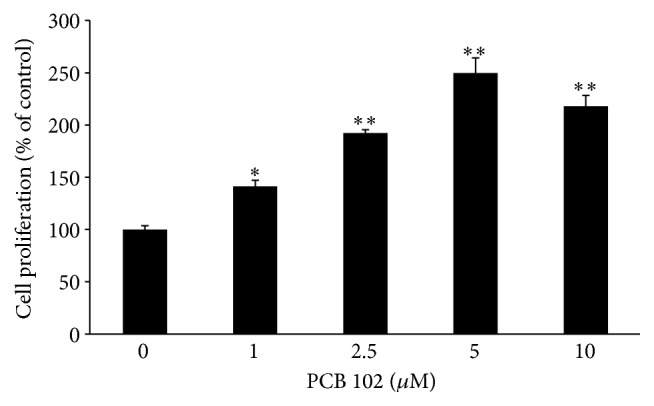
PCB 102 increases MCF-7/BOS breast cancer cell proliferation. MCF-7/BOS cells were treated with varying concentrations of PCB 102 (1, 2.5, 5, and 10 *μ*M) for 72 hours. Cell numbers were determined by hemocytometer and expressed as a percentage of the DMSO control (set at 100%). Values are expressed as mean ± SE (*n* = 3). Significance of differences between means: ^*∗*^ *P* < 0.05, ^*∗∗*^ *P* < 0.001 compared to DMSO control.

The growth of MCF-7/BOS cells was increased by PCB 102 in a dose-dependent manner (Figure 1). MCF-7/BOS cells were incubated with 1, 2.5, 5, and 10 *μ*M of PCB 102 for 72 hours. Cell proliferation was determined by hemocytometer. 1 *μ*M of PCB 102 elicited approximately a 40% increase in cell proliferation in comparison to the DMSO-treated control cells, whereas 5 *μ*M of PCB 102 induced the highest stimulation of cell proliferation (250% of control).

### 3.2. Effect of ER Antagonists on PCB 102-Mediated Cell Proliferation

**Figure 2:**
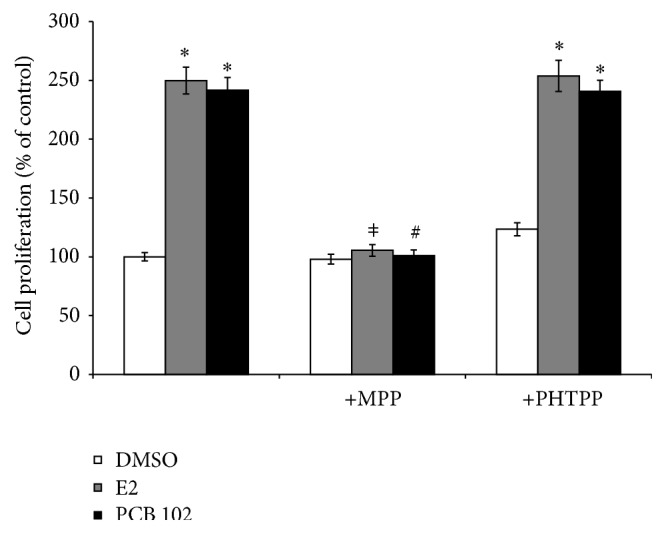
PCB 102-induced cell proliferation is mediated by ER*α*. MCF-7/BOS cells were treated with 1 nM E2 or 5 *μ*M PCB 102 with and without the ER*α*-specific inhibitor MPP (1 *μ*M) or the ER*β*-specific inhibitor PHTPP (1 *μ*M) for 72 hours. Cell numbers were determined by hemocytometer and expressed as a percentage of the DMSO control (set at 100%). Values are expressed as mean ± SE (*n* = 3). ^*∗*^Significantly different compared to control, *P* < 0.05; ^*ǂ*^significantly different compared to E2 treatment, *P* < 0.05; and ^#^significantly different compared to PCB treatment, *P* < 0.05.

Since some PCBs are known to exhibit estrogenic activity (reviewed in Discussion), we performed experiments to determine whether PCB 102-induced cell proliferation was estrogen receptor- (ER-) mediated. To address this question, we utilized two types of antiestrogens: MPP, an ER*α*-selective antagonist, and PHTPP, an ER*β*-selective antagonist. As shown in Figure 2, in the presence of PCB 102 (5 *μ*M) or E2 (1 nM), MCF-7/BOS cell proliferation increased to approximately 250% of control. This increase in cell proliferation was completely blocked with the addition of the ER*α*-specific inhibitor MPP (1 *μ*M), whereas the ER*β*-specific inhibitor PHTPP (1 *μ*M) did not inhibit PCB- or E2-induced cell proliferation, thus confirming the role of ER*α* rather than ER*β* in mediating the stimulatory effects of PCB 102 on cell proliferation.

### 3.3. Effect of EGCG Alone and in Combination with PCB 102 on MCF-7/BOS Breast Cancer Cell Proliferation

**Figure 3:**
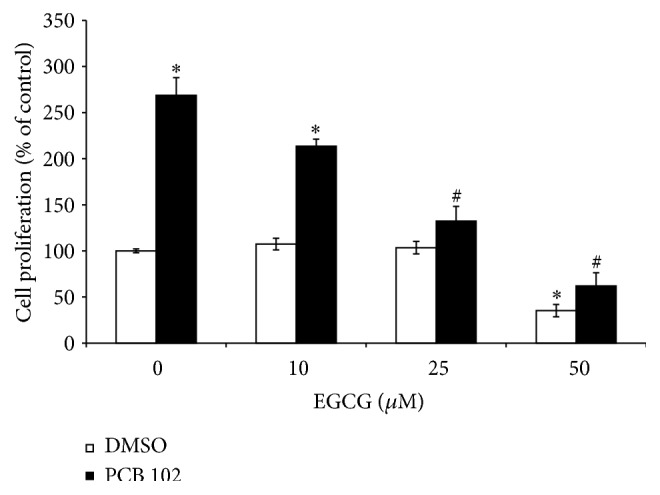
EGCG suppresses PCB 102-induced cell proliferation. MCF-7/BOS cells were treated with 5 *μ*M PCB 102 alone and in the presence of EGCG (10–50 *μ*M) for 72 hours. Cell numbers were determined by hemocytometer and expressed as a percentage of the DMSO control (set at 100%). Values are expressed as mean ± SE (*n* = 3). ^*∗*^Significantly different compared to control, *P* < 0.05; ^#^significantly different compared to PCB treatment, *P* < 0.05.

To determine whether EGCG can modulate MCF-7/BOS cell proliferation, cells were incubated with 10, 25, and 50 *μ*M EGCG alone for 72 hours. Neither 10 *μ*M EGCG nor 25 *μ*M EGCG alone had an effect on cell proliferation (Figure 3). In contrast, 50 *μ*M EGCG alone decreased cell growth approximately 65% without a reduction in overall cell viability. To determine if EGCG could suppress PCB 102-induced cell proliferation, cells were incubated with 5 *μ*M PCB 102 alone and in combination with 10, 25, or 50 *μ*M EGCG. The addition of EGCG caused a dose-dependent inhibition of PCB 102-induced cell proliferation, with nearly complete inhibition at 25 *μ*M.

### 3.4. Effect of EGCG and ER Antagonists on MCF-7/BOS Breast Cancer Cell Proliferation

Since PCB 102-induced cell proliferation was antagonized by EGCG and blocked by the ER*α*-specific inhibitor MPP, we evaluated the effect of EGCG in combination with the ER antagonists on MCF-7/BOS cell proliferation. We questioned whether the antiestrogens would block the decrease in cell proliferation mediated by 50 *μ*M EGCG. To address this issue, MCF-7/BOS cells were pretreated with 1 *μ*M MPP or 1 *μ*M PHTPP for 24 hours and then treated with 50 *μ*M EGCG for an additional 72 hours. Cells treated with 50 *μ*M EGCG alone were pretreated with DMSO for 24 hours and then treated with 50 *μ*M EGCG for an additional 72 hours.

**Figure 4:**
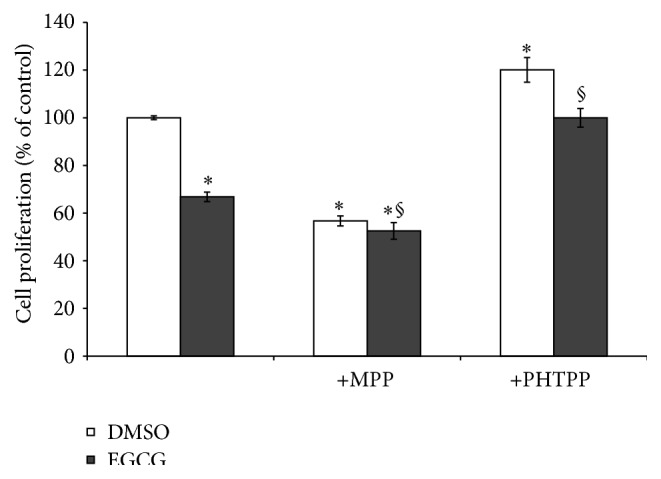
ER*β*-specific inhibitor PHTPP blocks inhibition of cell proliferation by EGCG. MCF-7/BOS cells were pretreated with the ER*α*-specific inhibitor MPP (1 *μ*M) or the ER*β*-specific inhibitor PHTPP (1 *μ*M) for 24 hours and then treated with 50 *μ*M EGCG for an additional 72 hours. Cell numbers were determined by hemocytometer and expressed as a percentage of the DMSO control (set at 100%). Values are expressed as mean ± SE (*n* = 3). ^*∗*^Significantly different compared to control, *P* < 0.05; ^§^significantly different compared to treatment with EGCG alone, *P* < 0.05.

As a result of the pretreatments, the cells grew for 48 hours prior to EGCG exposure and thus 50 *μ*M EGCG alone in this set of experiments decreased cell growth approximately 34% (Figure 4). Also as a result of the pretreatments, the cells were exposed to the ER*α*-specific inhibitor MPP (1 *μ*M) and the ER*β*-specific inhibitor PHTPP (1 *μ*M) for a total of 96 hours in this set of experiments. A time-dependent inhibition of cell proliferation was observed for cells exposed to ER*α*-specific inhibitor MPP alone; the 96-hour exposure resulted in a 44% decrease in cell proliferation compared to the control. In contrast, cells exposed to the ER*β*-specific inhibitor PHTPP alone for 96 hours resulted in a 20% increase in cell proliferation compared to the control. Importantly, the antiproliferative effects of 50 *μ*M EGCG on MCF-7/BOS cells were effectively abrogated by the ER*β*-specific inhibitor PHTPP but not by the ER*α*-specific inhibitor MPP.

## 4. Discussion

In this study, we evaluated the effects of PCB 102 and the green tea catechin EGCG, individually and in combination, on cell proliferation in estrogen-sensitive MCF-7/BOS breast cancer cells. PCB 102, found in the commercially used PCB mixture Aroclor 1242, was selected for our study because Aroclor 1242 is a major source of local contamination in the Fox River, Wisconsin, due to use by the paper industry in this region [31]. We found that PCB 102 stimulated cell proliferation in MCF-7/BOS breast cancer cells in a dose-dependent manner. The proliferative effects of PCB 102 were mediated by ER*α*, a finding consistent with what has been observed with other PCBs exhibiting estrogenic activity [15, 32], and could be blocked with the ER*α*-specific inhibitor MPP but not with the ER*β*-specific inhibitor PHTPP. To our knowledge, this is the first report to show that PCB 102 exhibits estrogenic activity. Additionally, the proliferative effects of E2 in MCF-7/BOS cells (used as positive control) were completely abrogated by the selective ER*α* antagonist MPP in MCF-7/BOS cells, as observed previously in normal mammary epithelial cells, PC3 prostate cancer cells, and MCF-7 breast cancer cells [33–35].

Interestingly, the proliferative effects of PCB 102 via ER*α* could also be antagonized by the green tea catechin EGCG. We found that EGCG caused a dose-dependent inhibition of PCB 102-induced cell proliferation, with nearly complete inhibition at 25 *μ*M EGCG. Green tea extract and EGCG, the major catechin in green tea, both suppress the activity of estrogen via ER*α* and block ER*α*-dependent transcription [36, 37]. Our results suggest that EGCG also has the ability to suppress the activity of environmental estrogens. Similar findings have been reported for the phytoestrogens genistein and luteolin, which suppress the estrogenic activity of the industrial environmental estrogens alkylphenols and bisphenol A (BPA), respectively [38].

Previous studies have shown that EGCG inhibits breast cancer cell proliferation by downregulating cyclin and CDK expression, as well as inducing expression of the CDK inhibitors p21 and p27, thereby triggering G1 cell cycle arrest [39, 40]. Sensitivity to EGCG treatment is highly dependent on cell type [24, 41], and, in some cancer cell lines, high concentrations of EGCG (≥85 *μ*M) induce apoptosis [41, 42]. In our studies with MCF-7/BOS cells, treatment with EGCG alone did not inhibit cell growth until the highest concentration of 50 *μ*M was reached. Our results are similar to other studies using MCF-7 cells in which 40–50 *μ*M EGCG treatment for 72 hours resulted in inhibition of proliferation without a significant decrease in cell viability [42–44]. In addition, we found that the susceptibility to EGCG treatment alone in MCF-7/BOS cells was affected by the timing of EGCG exposure after plating. Adding 50 *μ*M EGCG alone to cells 24 hours after plating (as used for the PCB ± EGCG cotreatment experiments) resulted in a 65% decrease in proliferation, whereas adding the same concentration to MCF-7/BOS cells 48 hours after plating (as used for the EGCG ± ER antagonists experiment due to 24-hour pretreatment with antagonists) resulted in a 34% decrease in proliferation. A possible explanation for this difference in EGCG susceptibility after plating may be the fact that the cells exposed at a later time are physiologically more stable.

While the concentrations of EGCG (10–50 *μ*M) used in our experiments are within the same range as many other short-term cell culture studies, the concentrations exceed the average maximum plasma concentration of 8 *μ*M detected in humans after oral administration of EGCG [45]. However, long-term culture of breast cancer cells with 8 *μ*M EGCG also resulted in growth inhibition [46]. Furthermore, sera from breast cancer patients given EGCG orally for several weeks in combination with radiotherapy inhibited* in vitro* cultures of metastatic breast cancer cells [47]. Together these findings support the chemoprotective potential of EGCG* in vivo.*

Although EGCG is able to bind to both ER isoforms, since the 3-gallate group mimics the 7*α*-position of E2 [48], it has a greater binding affinity for ER*β* than ER*α* [23]. MCF-7/BOS cells express both ER*α* and ER*β*; however, ER*α* is the predominant ER type expressed [49]. In cells expressing both receptor types, ER*β* acts as a dominant repressor of ER*α* function; thus ER*β* negatively modulates ER*α*-mediated transcriptional activity [50–52] and inhibits ER*α*-mediated breast cancer cell proliferation and tumor formation [51, 53, 54]. Given the protective effect of ER*β*, due to its ability to counteract ER*α* activity, we wanted to investigate whether ER*β* plays an important role in mediating inhibition of cell proliferation by 50 *μ*M EGCG alone. The antiproliferative action of EGCG alone in MCF-7/BOS cells was blocked by the ER*β*-specific inhibitor PHTPP, but not by the ER*α*-specific inhibitor MPP; thus our results indicate that EGCG is most likely acting as an ER*β* agonist to inhibit cell proliferation. EGCG has been previously shown to act as an ER*β* agonist in MCF-7 cells at the same concentrations used in our study (10–50 *μ*M) [23]. Other phytoestrogens have also been shown to preferentially bind [55] and utilize ER*β* over ER*α* to modulate transcriptional activity and cell proliferation [56]. The antiproliferative action of the flavonoid apigenin in prostate and breast cancer cells is mediated by ER*β* [57] as are the antiproliferative effects of soy isoflavones in colon cancer cells [58] and the phytoestrogen farrerol in vascular smooth muscle cells [59]. In all cases, inhibition of cell proliferation was reversed by using the ER*β*-specific antagonist PHTPP or siRNA knockdown of ER*β* but not by the ER*α*-specific antagonist MPP or siRNA knockdown of ER*α* [57, 59].

We also found that inhibition of ER*β*, using the ER*β*-specific antagonist PHTPP, resulted in a significant increase in MCF-7/BOS cell proliferation even in the absence of E2. A similar effect has been observed in normal mammary epithelial cells and T47D and MCF-7 breast cancer cells [33, 60, 61]. In contrast, inhibition of ER*α*, using the ER*α*-specific inhibitor MPP, for an extended amount of time (96 hours) resulted in a significant decrease in MCF-7/BOS cell proliferation. These results were in accordance to those obtained previously with normal mammary epithelial cells [33]. Together these findings suggest that cell proliferation in MCF-7/BOS cells is influenced by the ER*α*/ER*β* ratio. Thus the ability of EGCG to mediate its antiproliferative action via ER*β* and modify the estrogenic activity of industrial environmental estrogens via ER*α* may contribute to its role as a dietary chemopreventive agent.

While the focus of this study is on the ability of EGCG to suppress the proliferative effects of PCBs in estrogen responsive cells, EGCG may also be able to counteract the effects of PCBs in estrogen receptor negative (ER^−^) breast cancer cells. PCBs induce overexpression of vascular endothelial growth factor (VEGF) [62], enhance cell migration, and promote metastasis in ER^−^ breast cancer cells [63]. In contrast, EGCG inhibits VEGF expression, migration, and invasion of ER^−^ breast cancer cells [64]. In addition, breast cancer patients given EGCG orally plus radiotherapy showed significantly lower serum levels of VEGF and reduced activity of invasion promoting metalloproteinases [47] compared to patients who received radiotherapy alone. Thus the ability of EGCG to counteract the detrimental effects of PCBs in ER^−^ breast cancer cells warrants further investigation.

## 5. Conclusion

Our results demonstrate that PCB 102 exhibits estrogenic activity and induces cell proliferation in estrogen-sensitive MCF-7/BOS breast cancer cells, via ER*α*, in a dose-dependent manner. Furthermore, this study demonstrates that EGCG can suppress the estrogenic activity of polychlorinated biphenyls, specifically PCB 102, in breast cancer cells* in vitro*. The antiproliferative action of EGCG was blocked by the ER*β*-specific inhibitor PHTPP; thus the results suggest that EGCG can act as an ER*β* agonist to induce growth inhibition. These findings are significant since they indicate that EGCG has the ability to mitigate the detrimental effects of industrial environmental estrogens in human breast cancer cells. Moreover they further illustrate how consumption of green tea may play an important role in breast cancer chemoprevention.
